# Validity of EQ-5D-5L health-related quality of life questionnaire in self-reported diabetes: evidence from a general population survey

**DOI:** 10.1186/s12955-021-01780-2

**Published:** 2021-05-05

**Authors:** Agnieszka Jankowska, Katarzyna Młyńczak, Dominik Golicki

**Affiliations:** 1 National Institute of Cardiology, Warsaw, Poland; 2 Department of Experimental and Clinical Pharmacology, Medical University of Warsaw, Banacha 1b St, 02-097 Warsaw, Poland

**Keywords:** EQ-5D-5L, Health-related quality of life, Patient-reported outcomes, Psychometrics, Diabetes Mellitus

## Abstract

**Background:**

This study aimed to assess the validity of the EQ-5D-5L in respondents with self-reported diabetes coming from a representative general population survey.

**Methods:**

2974 respondents from the general adult population of Poland, chosen with multi-stage random sampling, were surveyed with HRQoL instruments (EQ-5D-5L, EQ VAS, SF-12, EQ-5D-3L) and a screening question about diabetes. To obtain EQ-5D index values, we used country-specific Polish value sets. We compared the instruments in terms of the ceiling effect, discriminatory power and frequency of individual health states. We evaluated construct validity in terms of known-groups validity and convergent validity of EQ-5D-5L dimensions and index values with other HRQoL measures.

**Results:**

In respondents with diabetes (n = 247), the percentage reporting 'no problems' with EQ-5D-3L was reduced by 34.5% with the use of EQ-5D-5L (from 14.2% to 9.3%, respectively). A significant improvement in informativity was noticed in mobility and pain/discomfort dimensions (a relative increase of 23.1% and 22.7%, respectively). Known-groups construct validity analysis confirmed prior hypotheses—index scores were higher in the following groups: younger respondents, males, those taking no medication or oral antidiabetic drugs, and respondents with higher levels of education. The convergence between related EQ-5D-5L and EQ-5D-3L or SF-6D dimensions was stronger than between unrelated dimensions. The Bland–Altman analysis showed a mean difference between EQ-5D-5L and EQ-5D-3L, SF-6D, EQ VAS/100 index scores of 0.047, 0.165 and 0.231 respectively.

**Conclusions:**

Our results support the validity of the EQ-5D-5L descriptive system and EQ-5D-5L index, based on the directly measured value set in respondents with self-reported diabetes coming from the general population.

**Supplementary Information:**

The online version contains supplementary material available at 10.1186/s12955-021-01780-2.

## Background

Diabetes mellitus leads to severe micro- and macrovascular complications and results in increased mortality. Complications and treatment of the disease reduce health-related quality of life (HRQoL) [1, 2]. This effect can be measured with numerous disease-specific questionnaires, such as the Diabetes Health Profile (DHP) [3], Diabetes Quality of Life measure (DQOL) [4], Diabetes-39 (D-39) [5] or the Audit of Diabetes Dependent Quality of Life (ADDQoL) [6]. Generic quality of life instruments, such as the 36-Item Short-Form Health Survey (SF-36), the World Health Organization Quality-of-Life Scale (WHOQOL-BREF) or the EQ-5D questionnaire, may complement this measurement or be used separately as a standalone measure [7].

The three-level version of EQ-5D is commonly used in diabetes research and for modelling health outcomes in economic evaluations of antidiabetic drugs. Multiple studies have confirmed EQ-5D-3L measurement properties in patients with diabetes [8]. Recent years have brought the development of a new five-level version of EQ-5D, intended to improve some psychometric properties of the original three-level version [9]. Early assessment within a multi-country study involving patients with eight chronic conditions demonstrated several advantages of the new version: a reduced ceiling effect, improved discriminatory power and convergent validity [10]. These findings were confirmed by a recent review [11].

Several reports on the validity of the EQ-5D-5L in patients with diabetes were published [10, 12–15]. The majority of them focused on type 2 diabetes [12, 14, 15]. Analyses also confirmed the reliability [13, 14] and responsiveness [15] of the EQ-5D-5L in the clinical context under evaluation. One of the studies employed a qualitative examination of the content validity [12]. In published research, the EQ-5D-5L index was not estimated (only EQ-5D descriptive results were presented) [10, 12] or the EQ-5D-5L index was based on mapping with EQ-5D-3L index values (cross-over value set) [13, 14]. The only EQ-5D-5L validation study in patients with diabetes with health state utility values based on the directly elicited set come from Alberta province (Canada) and used a Canadian time trade-off-based value set [15]. Reports presented comparisons with EQ-5D-3L [13], SF-36 [13] and SF-6D [15]. In none of the studies was convergent validity with SF-12 domains examined. There is also no comparison based on direct methods of the EQ-5D-5L index with EQ-5D-3L, nor with the EQ-5D-5L-index based on a crosswalk algorithm (EQ-5D-5L_crosswalk_ index).

Our study aimed to assess the validity of the EQ-5D-5L questionnaire in respondents with self-reported diabetes coming from a general population survey. We aimed to perform a comparison with EQ-5D-3L, SF-12, SF-6D, EQ VAS and the EQ-5D-5L_crosswalk_ index.

## Methods

### Respondents

Adult Polish citizens, participants of a nationally representative general population survey [16, 17] who confirmed having a diagnosis of diabetes and had complete HRQoL data, were allowed to enter the validation study.

General population survey sample recruitment and interviewing was carried out by a market research company—the Public Opinion Research Centre (CBOS). To obtain a representative study group, multi-stage random sampling was used. Firstly, the Polish adult population was divided into 65 strata, taking into account the country's administrative division into 16 provinces and the type and size of the localities where participants resided. The pre-determined study sample was proportionally allocated into layers in a way reflecting the general population structure. Multi-stage random sampling was performed in three steps: first–localities (towns/cities or villages), second—small areas (one or several adjacent streets), third—eight people living in separate households from each of the selected areas. The final selection of individuals was based on their Personal Identification Number (PESEL) [16, 18].

Respondents were classified as having self-reported diabetes if, in response to the following question: "Have you ever been diagnosed with diabetes?", they chose one of the following answers: (a) "Yes, but I don't take any medication", (b) "Yes, I take antidiabetic medication (other than insulin)" or (c) "Yes, I take insulin". Respondents on combined antidiabetic treatment were allowed to choose both answer (b) and (c). The diagnosis was not verified using blood HbA1c, fasting plasma glucose level or using medical records or registries.

### Measures

Data were collected during face-to-face interviews led by professional CBOS interviewers in participants' homes. The health-related quality of life of respondents was measured with: EQ-5D-5L [9], EQ VAS, SF-12v2 [19] and EQ-5D-3L [20, 21]. Questionnaire instruments were always presented in the same order as mentioned above. Self-completed paper and pencil versions were used. Sociodemographic data covering age, sex, type and size of locality, administrative region, education level, professional status, religiosity and smoking status were collected using a computer-assisted personal interviewing (CAPI) system.

To obtain EQ-5D index values, we used three different country-specific Polish value sets: (1) a directly elicited EQ-5D-5L set, based on a hybrid model (combining time trade-off (TTO) and discrete choice experiment (DCE) data) [22], (2) an EQ-5D-5L set, based on mapped EQ-5D-3L values and official EuroQol Group crosswalk methodology [23, 24], and (3) a directly elicited EQ-5D-3L set, based on TTO [25]. As there is no Polish country-specific SF-6D value set, we used an SF-12v2-based algorithm for the United Kingdom, developed by Brazier et al. [26]. For comparative purposes, we presented all EQ VAS values transformed to a scale from 0 to 1 (divided per 100). The study was approved by the ethics committee of the Medical University of Warsaw. All participants gave informed consent before inclusion.

### Analysis

Only respondents with complete HRQoL data were included in the psychometric analysis. We analysed the proportion and level of logical inconsistencies in EQ-5D-5L–EQ-5D-3L pairs of answers according to a method proposed by Janssen et al. (see [10] for details). In short, an inconsistent response was defined as an EQ-5D-3L response followed by an EQ-5D-5L response that was two levels apart (grade 1 of inconsistency), three levels apart (grade 2 of inconsistency) or four levels apart (grade 3 of inconsistency). We compared EQ-5D-5L, EQ-5D-3L and SF-6D in terms of the frequency of individual health states, ceiling effect and informativity (the discriminatory power) [27]. We evaluated construct validity in terms of known-groups validity and convergent validity of EQ-5D-5L dimensions with SF-12 domains, SF-6D or EQ-5D-3L dimensions, as well as the convergence of EQ-5D-5L index with EQ-5D-3L, SF-6D indexes and EQ VAS.

Discriminatory power was assessed with the Shannon Index (*H'*), representing the absolute amount of captured informativity, and the Shannon Evenness Index (*J'*), reflecting the rectangularity of distribution regardless of the number of levels (for details, see [10]). When the instrument achieves an evenness of the distribution (rectangularity), *H'* approximates 2.32 (for EQ-5D-5L) or 1.58 (for EQ-5D-3L). At the same time, *J'* approaches 1.0, which indicates maximum informativity captured by the instrument [28].

Known-groups validity was determined for the EQ-5D-5L, EQ-5D-5L_crosswalk_, EQ-5D-3L and SF-6D indices regarding age group, sex, type of diabetes treatment, education level and subjective health status, as determined by the EQ VAS quartile [29, 30]. We hypothesized that health state utility would be higher in younger age groups, males, patients taking no medication or oral antidiabetic drugs, respondents with a medium or high level of education and with a superior subjective assessment of health.

Convergent validity was evaluated by examining the strength of association between the EQ-5D-5L and EQ-5D-3L dimensions, and between the EQ-5D-5L and SF-12 domains using a Spearman rank correlation. We used the following criteria to interpret strength of correlation: Rho < 0.20: absent, 0.20–0.34: poor, 0.35–0.50: moderate, > 0.50: strong [31, 32]. Additionally, the convergence of index values of the generic questionnaires, the SF-12 summary scores and EQ VAS were also assessed with Spearman rank correlation using the interpretation criteria mentioned above.

The relationships between instruments were explored with an intra-class correlation coefficient (ICC; one-way random effects model) and illustrated with Bland–Altman plots. These plots show the relationship between the means of scores (X-axis) and the differences between scores (Y-axis). The 95% limits of agreement were estimated using the following formula: mean of the differences (*d*) ± 1.96 × SD of *d* [33]. Differences lying within the 95% limits of agreement are usually interpreted as not clinically important and show that the two measurement methods could be used interchangeably. Potential proportional bias was investigated with linear regression. To examine the influence of scale range differences on observed differences in health state values, we ran an additional Bland–Altman analysis with all utility instruments adjusted to the same scale (from 0 to 1). All data analyses were performed with StatsDirect software (ver. 2.8.0).

## Results

From April 2014 to June 2014, 2974 respondents from the general population of Poland were surveyed with HRQoL instruments and a screening question about diabetes [14]. 255 (8.6%) individuals self-reported diagnosis of diabetes. Within this group, 247 (96.9%, mean age 64.6 years, 53.4% female) respondents had complete HRQoL data and were included in the psychometric analysis.

The overall proportion of inconsistent EQ-5D-5L responses, in comparison with EQ-5D-3L, was 7.9%, ranging from 4.9% for pain/discomfort to 14.2% for usual activities. The majority of inconsistencies (89.7%) were level 1, as defined by Janssen et al. [10].

**Fig. 1:**
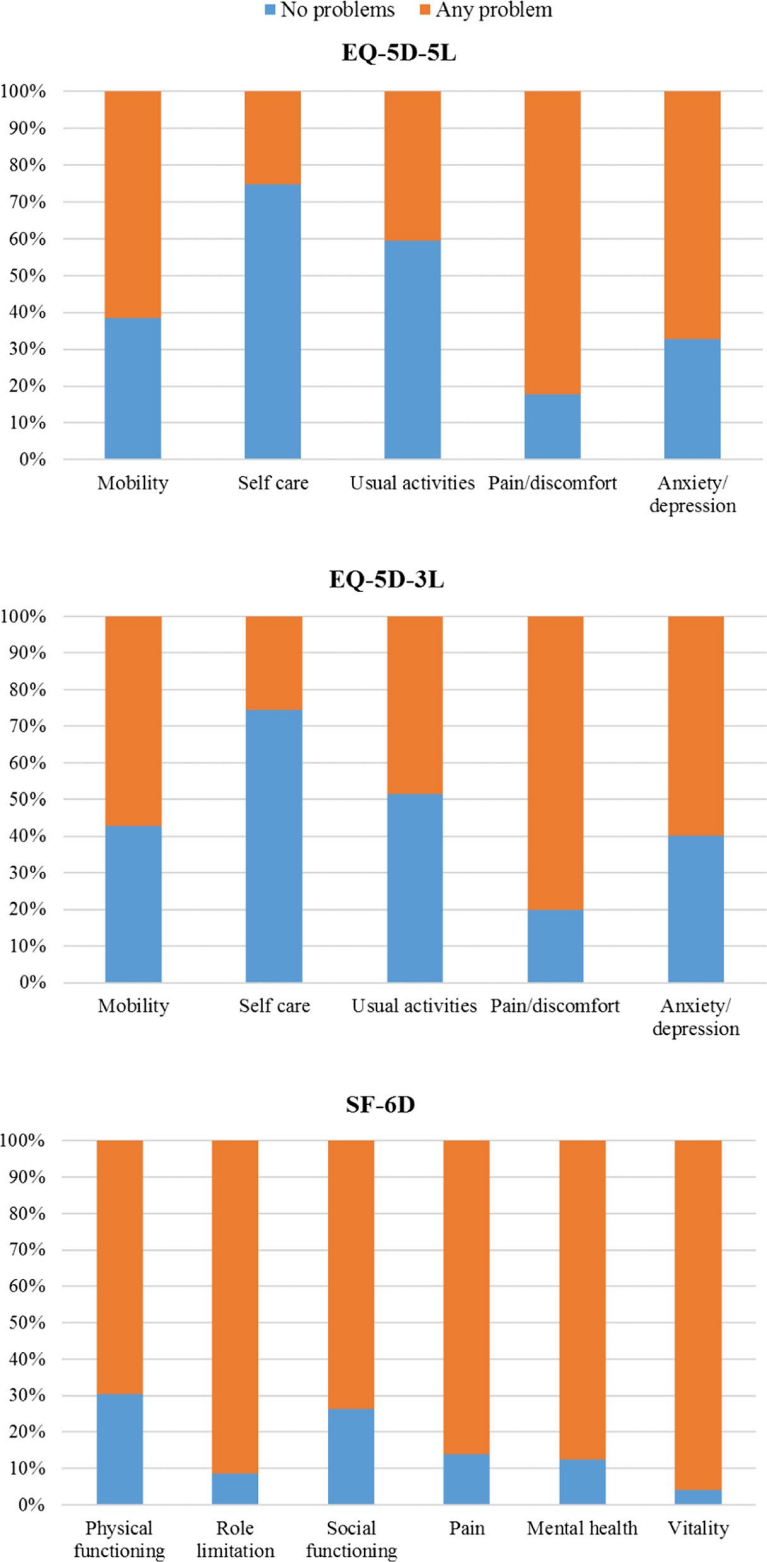
Response distribution of EQ-5D-5L, EQ-5D-3L and SF-6D domains

The proportion of respondents reporting 'no problems' was 14.2% for EQ-5D-3L and 9.3% for EQ-5D-5L (compared to 1.6% for SF-6D and 2.4% for EQ VAS). The relative reduction of the ceiling effect in EQ-5D-5L in comparison to EQ-5D-3L (34.5%) was highest in the anxiety/depression dimension (18.2%), followed by mobility (10.4%) and pain/discomfort (10.2%). However, within the usual activities domain, we noticed a relative increase of the ceiling effect (by 15.7%). Figure 1 shows the dichotomized response distributions of the EQ-5D-5L, EQ-5D-3L and SF-6D instruments.

Both the Shannon Index and Shannon Evenness Index showed high informativity of the EQ-5D-5L pain/discomfort and mobility dimensions in respondents with self-reported diabetes (Table 1). Moreover, the domains mentioned above showed the most significant improvement in relative discriminatory power, when moving from EQ-5D-3L to EQ-5D-5L (an increase in *J'* of 22.7% and 23.1% respectively). However, the change in the number of levels (from three to five) also resulted in a deterioration of relative informativity within the usual activities and self-care dimensions (a relative decrease in *J'* of 11.5% and 11.4% respectively).

**Table 1:** Shannon index (*H’*) and Shannon Evenness index (*J’*) for EQ-5D-5L and EQ-5D-3L

	*H'*		*J'*			
	EQ-5D-5L	EQ-5D-3L	EQ-5D-5L	EQ-5D-3L	Difference (5L − 3L)	%
Mobility	2.08	1.15	0.89	0.73	0.17	23.1
Self-care	1.25	0.96	0.54	0.61	− 0.07	− 11.4
Usual activities	1.68	1.30	0.73	0.82	− 0.09	− 11.5
Pain/discomfort	2.08	1.16	0.90	0.73	0.17	22.7
Anxiety/depression	1.92	1.24	0.83	0.79	0.04	5.5

^*^H'_*max*_ (EQ-5D-5L) = 2.32; *H'*_*max*_ (EQ-5D-3L) = 1.58

The total number of unique health states was 119 for EQ-5D-5L (most common 11111, n = 23 and 11122, n = 17), 43 for EQ-5D-3L (most common 11111, n = 35 and 22222, n = 29) and 172 for SF-6D (most common 243333, n = 8 and 242323, n = 7).

**Fig. 2:**
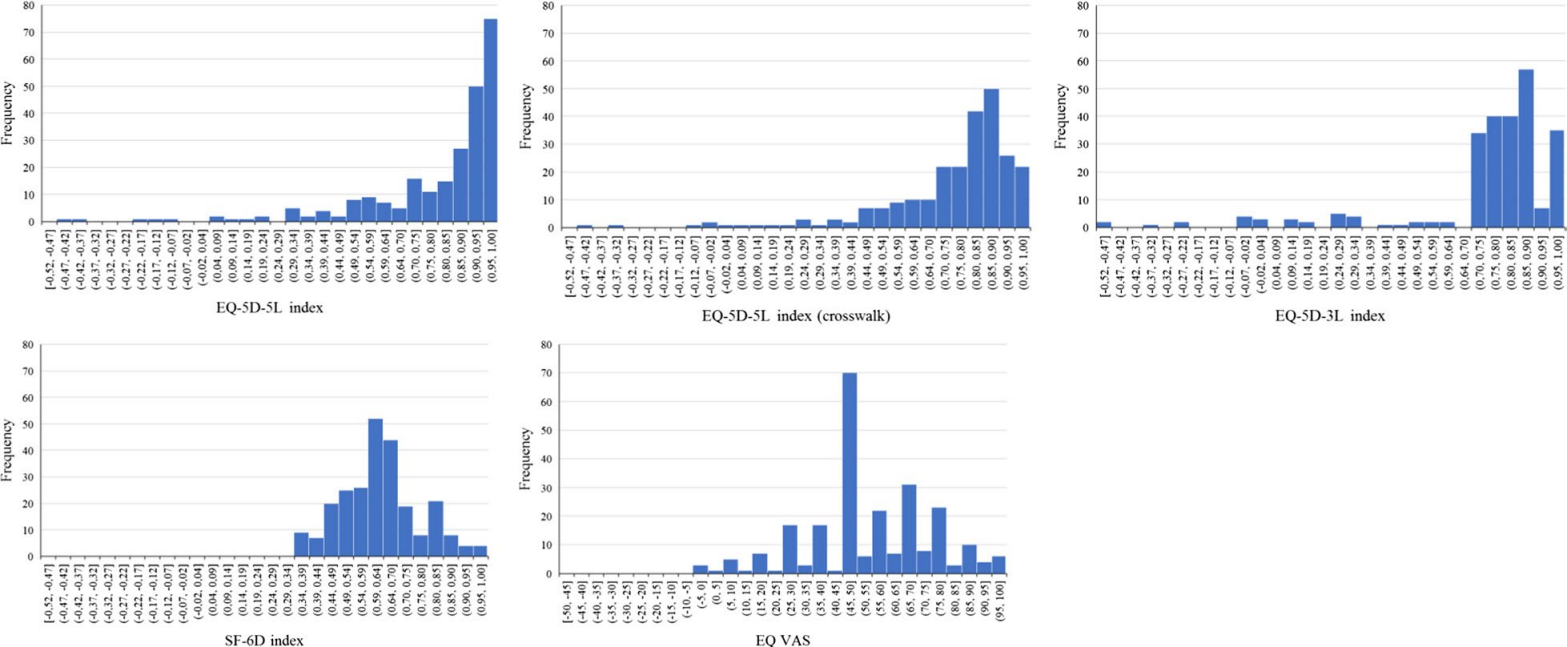
Distribution of four health status indices: EQ-5D-5L, EQ-5D-5L_crosswalk_, EQ-5D-3L, SF-6D and EQ VAS

The mean health state utility value for all the respondents with self-reported diabetes was highest when based on EQ-5D-5L—0.798 (SD 0.251; range − 0.446 to 1.0). The corresponding scores for the EQ-5D-5L_crosswalk_ index, EQ-5D-3L and SF-6D were lower by 0.044, 0.047 and 0.165 respectively. Figure 2 shows the distribution of scores for the four analysed health state utility instruments and EQ VAS.

The results for known-groups construct validity confirmed our prior hypotheses: index scores were higher in younger groups, males, those taking no medication or oral antidiabetic drugs, respondents with a medium or high level of education and respondents with better subjective assessment of health according to EQ VAS (Table 2). There were two unexpected outcomes: a lower utility level in patients with insulin therapy, in comparison to patients on combination treatment (for all instruments except SF-6D) and, in terms of EQ-5D-3L, identical scores were observed for the 18–50 and 51–60 age groups. Caution should be taken in the interpretation of known-groups validity results, as the majority of the observed differences were not statistically significant. Moreover, the sample size of the group with the combined treatment was limited.

**Table 2:** Known-groups construct validity: mean index-based scores of EQ-5D-5L, EQ-5D-5L_crosswalk_, EQ-5D-3L, SF-6D and EQ VAS (and 95% confidence intervals) by patient characteristics

	N (%)	Mean (95%CI)				
		EQ-5D-5L	EQ-5D-5L_crosswalk_	EQ-5D-3L	SF-6D	EQ VAS
All	247 (100.0)	0.798 (0.766, 0.829)	0.754 (0.725, 0.783)	0.751 (0.717, 0.785)	0.633 (0.616, 0.649)	0.567 (0.541, 0.594)
Age (years)						
18–50	26 (10.5)	0.883 (0.807, 0.958)	0.846 (0.774, 0.917)	0.836 (0.732, 0.940)	0.728 (0.667, 0.789)	0.675 (0.572, 0.778)
51–60	44 (17.8)	0.862 (0.804, 0.920)	0.815 (0.764, 0.867)	0.836 (0.782, 0.891)	0.643 (0.608, 0.679)	0.613 (0.548, 0.679)
61–70	103 (41.7)	0.825 (0.777, 0.872)	0.771 (0.727, 0.816)	0.744 (0.690, 0.798)	0.637 (0.612, 0.662)	0.572 (0.533, 0.611)
> 70	74 (30.0)	0.693 (0.627, 0.758)	0.662 (0.604, 0.720)	0.680 (0.610, 0.751)	0.586 (0.558, 0.614)	0.495 (0.452, 0.538)
Sex						
Female	132 (53.4)	0.772 (0.722, 0.822)	0.730 (0.685, 0.776)	0.729 (0.677, 0.781)	0.618 (0.595, 0.640)	0.546 (0.509, 0.583)
Male	115 (46.6)	0.828 (0.792, 0.864)	0.782 (0.749, 0.815)	0.777 (0.733, 0.820)	0.650 (0.625, 0.674)	0.591 (0.553, 0.630)
Diabetes treatment						
No medication	59 (23.9)	0.873 (0.818, 0.929)	0.827 (0.774, 0.881)	0.813 (0.749, 0.878)	0.669 (0.634, 0.704)	0.631 (0.577, 0.686)
OAD	120 (48.6)	0.795 (0.749, 0.842)	0.749 (0.707, 0.792)	0.756 (0.707, 0.804)	0.634 (0.609, 0.658)	0.575 (0.537, 0.613)
Insulin	55 (22.3)	0.727 (0.655, 0.799)	0.690 (0.627, 0.754)	0.672 (0.590, 0.754)	0.601 (0.567, 0.635)	0.493 (0.438, 0.548)
Combined treatment	13 (5.3)	0.781 (0.652, 0.910)	0.738 (0.630, 0.845)	0.760 (0.615, 0.905)	0.590 (0.531, 0.649)	0.519 (0.418, 0.621)
Education level						
Primary	58 (23.5)	0.696 (0.622, 0.771)	0.658 (0.592, 0.724)	0.643 (0.552, 0.735)	0.575 (0.543, 0.607)	0.499 (0.445, 0.553)
Secondary	146 (59.1)	0.817 (0.776, 0.858)	0.772 (0.734, 0.810)	0.774 (0.733, 0.815)	0.643 (0.621, 0.665)	0.584 (0.549, 0.619)
High	43 (17.4)	0.871 (0.823, 0.918)	0.824 (0.782, 0.867)	0.818 (0.758, 0.877)	0.674 (0.636, 0.711)	0.603 (0.542, 0.663)
EQ VAS						
0–24	17 (6.9)	0.311 (0.091, 0.531)	0.310 (0.111, 0.510)	0.195 (− 0.049, 0.438)	0.490 (0.447, 0.534)	0.124 (0.084, 0.163)
25–49	39 (15.8)	0.665 (0.595, 0.734)	0.635 (0.578, 0.692)	0.648 (0.563, 0.733)	0.516 (0.486, 0.546)	0.350 (0.333, 0.367)
50–74	136 (55.1)	0.847 (0.819, 0.875)	0.795 (0.770, 0.820)	0.791 (0.761, 0.822)	0.642 (0.624, 0.659)	0.572 (0.557, 0.586)
75–100	55 (22.3)	0.922 (0.880, 0.964)	0.876 (0.831, 0.920)	0.896 (0.863, 0.930)	0.736 (0.701, 0.772)	0.848 (0.827, 0.869)

*OAD* oral antidiabetic drugs

The results for convergent validity of dimensions are shown in Table 3. The EQ-5D-5L and EQ-5D-3L dimensions revealed similar correlations, with a high likelihood of statistical insignificance of difference between the two. The SF-12 social functioning domain was, in general, poorly correlated with the EQ-5D-5L dimensions and poorly correlated or uncorrelated with the EQ-5D-3L dimensions (SC, UA and MO, PD, AD). The relationships between index scores are reported in Table 4. EQ-5D-5L index scores were strongly correlated with other index scores—EQ-5D-5L_crosswalk_, EQ-5D-3L and SF-6D. They were also strongly correlated with EQ VAS and physical component scores (PCS-12), but poorly correlated with mental component scores (MCS-12).

**Table 3:** Convergent validity with SF-12, SF-6D and EQ-5D-3L domains (Spearman’s rank correlation coefficients) (N = 247)

	EQ-5D-5L					EQ-5D-3L				
	MO	SC	UA	PD	AD	MO	SC	UA	PD	AD
SF-12										
PF	− 0.74	− 0.48	− 0.60	− 0.67	− 0.42	− 0.67	− 0.46	− 0.64	− 0.53	− 0.40
RP	− 0.69	− 0.47	− 0.61	− 0.67	− 0.41	− 0.64	− 0.51	− 0.61	− 0.52	− 0.36
BP	− 0.60	− 0.39	− 0.53	− 0.69	− 0.43	− 0.55	− 0.45	− 0.57	− 0.53	− 0.41
GH	− 0.57	− 0.45	− 0.51	− 0.65	− 0.52	− 0.51	− 0.47	− 0.59	− 0.53	− 0.50
VT	− 0.52	− 0.41	− 0.54	− 0.48	− 0.38	− 0.48	− 0.48	− 0.57	− 0.38	− 0.41
SF	0.20	0.22	0.22	0.23	0.20	0.12*	0.26	0.22	0.12*	0.16
RE	− 0.47	− 0.37	− 0.49	− 0.39	− 0.53	− 0.45	− 0.44	− 0.51	− 0.40	− 0.49
MH	− 0.37	− 0.29	− 0.37	− 0.36	− 0.58	− 0.35	− 0.30	− 0.35	− 0.34	− 0.58
SF-6D										
PF	0.69	0.51	0.60	0.68	0.42	0.62	0.46	0.61	0.51	0.41
RL	0.37	0.24	0.34	0.32	0.42	0.38	0.29	0.39	0.36	0.48
SF	0.49	0.42	0.53	0.52	0.48	0.45	0.43	0.51	0.48	0.47
PA	0.60	0.39	0.53	0.69	0.43	0.55	0.45	0.57	0.53	0.41
MH	0.39	0.25	0.35	0.36	0.51	0.37	0.28	0.39	0.35	0.51
VT	0.52	0.41	0.54	0.48	0.38	0.48	0.48	0.57	0.38	0.41
EQ-5D-3L										
MO	*0.79*	0.43	0.58	0.56	0.39					
SC	0.55	*0.75*	0.64	0.42	0.40					
UA	0.70	0.59	*0.73*	0.54	0.43					
PD	0.52	0.37	0.46	*0.69*	0.41					
AD	0.40	0.30	0.33	0.42	*0.73*					

*All correlations statistically significant except those marked with an asterisk. Cells with related EQ-5D-5L and EQ-5D-3L dimensions marked in italics. *AD* anxiety/depression; *BP* bodily pain; *GH* general health; *MH* mental health; *MO* mobility; *PA* pain; *PD* pain/discomfort; *PF* physical functioning; *RE* role emotional; *RL* role limitations; *RP* role physical; *SC* self-care; *SF* social functioning, *UA* usual activities; *VT* vitality

**Table 4:** Convergent validity with generic questionnaires indexes (Spearman's rank correlation coefficients) (N = 247)

	EQ-5D-5L index	EQ-5D-5L_crosswalk_ index	EQ-5D-3L index	SF-6D index	PCS-12	MCS-12	EQ VAS
EQ-5D-5L index	–	0.99	0.85	0.76	0.80	0.30	0.63
EQ-5D-5L_crosswalk_ index		–	0.87	0.75	0.79	0.31	0.62
EQ-5D-3L index			–	0.74	0.76	0.38	0.60
SF-6D index				–	0.73	0.54	0.63
PCS-12					–	0.06*	0.64
MCS-12						–	0.30
EQ VAS							–

^*^All correlations statistically significant except those marked with an asterisk. *MCS-12* Mental Component Summary score; *PCS-12* Physical Component Summary score

An ICC of 0.81 between the EQ-5D-5L and EQ-5D-3L index scores indicated good agreement. ICCs of 0.29 and 0.27 showed a poor agreement of EQ-5D-5L with SF-6D scores and EQ VAS, respectively. The Bland–Altman analysis showed a mean difference of 0.047 (95% limits of agreement: − 0.258 to 0.352) between the EQ-5D-5L and EQ-5D-3L index scores, a difference of 0.165 (-0.226 to 0.557) between EQ-5D-5L and SF-6D, and a difference of 0.231 (-0.183 to 0.644) between the EQ-5D-5L and EQ VAS index scores. EQ-5D-5L index scores were higher in 64.0%, 86.6% and 88.7% cases respectively. Overall, 6.4%, 4.9% and 4.4% observations were outside the 95% limits of agreement. The discrepancy between EQ-5D-5L and SF-6D index scores was larger for lower utility values. The adjusted Bland–Altman analysis showed an increase in the mean difference between EQ-5D-5L and SF-6D values to 0.395 (see Additional file 1: Fig. 1).

**Fig. 3:**
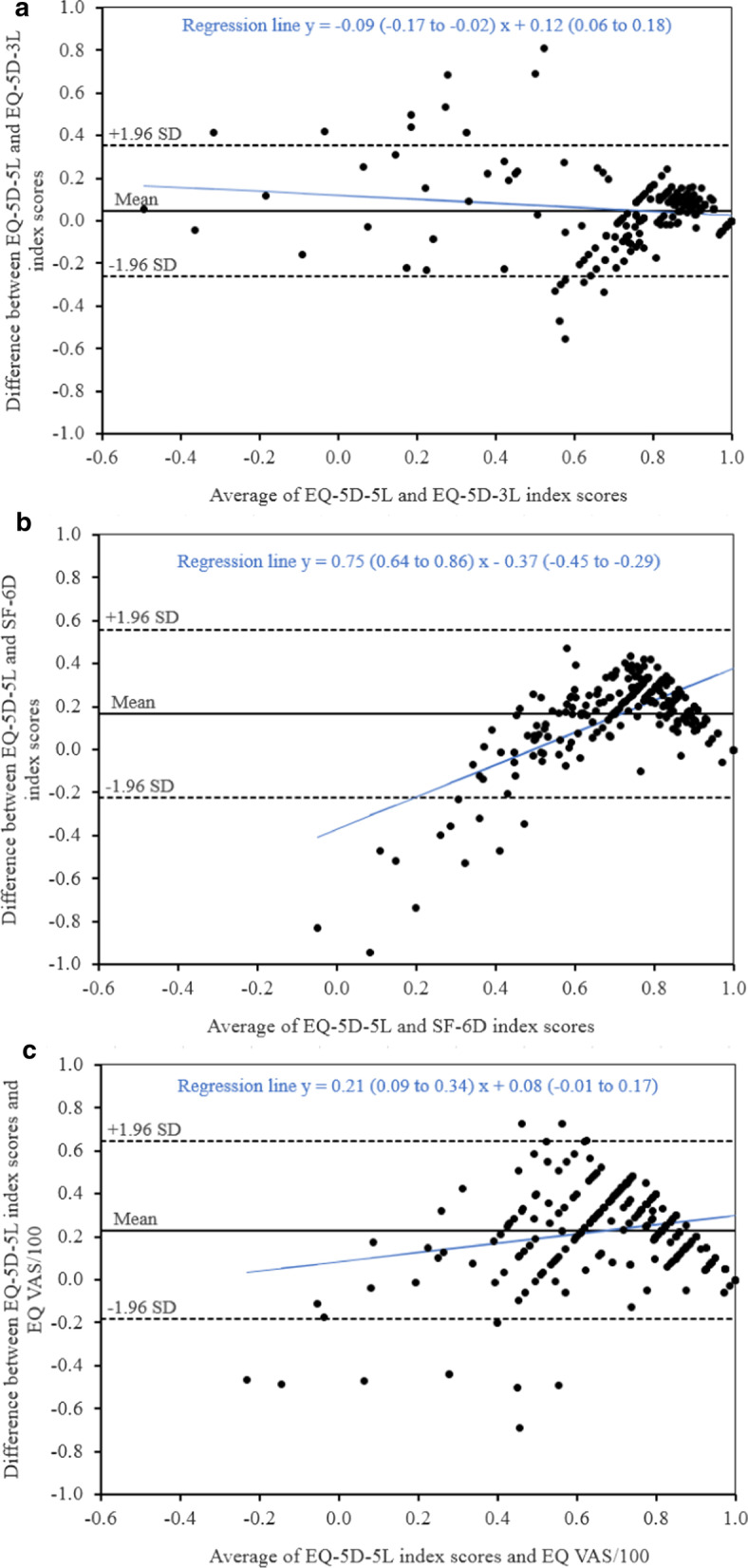
Bland–Altman plots of EQ-5D-5L and **a** EQ-5D-3L, **b** SF-6D and **c** EQ VAS scores (blue lines represent regression lines)

Linear regression analysis showed signs of proportional bias, indicating that the methods do not agree equally across the range of measurements (Fig. 3).

## Discussion

This study indicates that EQ-5D-5L index values based on a directly measured value set, EQ-5D-5L index values based on a crosswalk algorithm and EQ-5D-3L index values provide valid measurement in the population of Polish respondents with self-reported diabetes, coming from a general population survey. We confirmed the construct validity of the EQ-5D-5L questionnaire in terms of known-groups validity and convergence validity with other generic HRQoL measures—EQ-5D-3L, SF-12, SF-6D, EQ VAS and the EQ-5D-5L_crosswalk_ index. According to our best knowledge, this is the first study reporting convergent validity of the EQ-5D-5L and SF-12 descriptive systems in patients with diabetes. These are also the first comparisons between EQ-5D-5L index values based on a directly measured value set and the EQ-5D-5L_crosswalk_ index, and between EQ-5D-5L index values based on a directly measured value set and the corresponding values in EQ-5D-3L, in patients with diabetes.

It was surprising to find that in the analysed population of self-reported diabetes patients, three dimensions (MO, PD, AD) seemed to function better psychometrically in the EQ-5D-5L questionnaire, while the remaining two performed better in the EQ-5D-3L version. The dimensions of usual activities (UA) and self-care (SC) were characterized by an increase in the ceiling effect (by 15.7% and 0.5% respectively) and a decrease in relative discriminatory power (by 11.4% and 11.5% respectively) in the five-level EQ-5D, compared to the three-level version.

Although other authors of psychometric studies in diabetes have observed similar relationships, this was only to a limited extent. In a study by Pattanaphesaj et al., the EQ-5D-5L questionnaire demonstrated better severity level distribution than EQ-5D-3L in all dimensions other than self-care [13]. A separate study also demonstrated better distribution than EQ-5D-3L across all dimensions other than SC and PD [34] However, in both publications, SC had an overall low absolute informativity in patients with diabetes. This problem was also indicated in the qualitative study of Matza (2015), in which respondents stated that SC is the dimension having the lowest relevance to type 2 diabetes problems [12]. In a recent systematic review of EQ-5D-5L psychometric properties, Feng et al. found that SC is the dimension with the lowest percentage of reported problems across all of the disease groups and healthy samples under analysis [11]. A previous systematic review of these authors indicated that SC is also the domain in which a lower ceiling effect for EQ-5D-3L than EQ-5D-5L most commonly occurs (20% of studies included in the review) [35].

According to Gamst-Klaussen et al. (2018), SC and UA problems are a reflection of the other three dimensions. More specifically, PD and AD are causal indicators that derive SC and UA, with MO in the intermediate position [36]. Interestingly, in our study, the two dimensions with the most significant number of inconsistencies between the five-level and three-level descriptive systems were UA and SC (14.2% and 8.5% inconsistencies respectively). Perhaps to some extent, these results stemmed from the fixed order of presentation of the questionnaires (EQ-5D-5L first, then SF-12, and finally EQ-5D-3L). Self-care and usual activities are certainly less intuitive dimensions than pain, anxiety, or mobility problems. Respondents are generally less likely to report SC [37] and UA restrictions [16]. We can hypothesise that the presentation of the SF-12 questionnaire, preceding the completion of EQ-5D-3L, prompted the respondents to analyse their situation more thoroughly and thus become more aware of their limitations, resulting in a higher percentage of reported complaints (for UA and SC, in 3L than 5L) and a relatively high proportion of inconsistencies within these dimensions. In such a situation, our observation would only be an artefact of the survey design and the sequence of presentation of the questionnaires. The final confirmation can only be obtained from further psychometric studies in diabetic patients, employing a randomised sequence of questionnaires.

In our study, the Bland–Altman analysis showed a mean difference in utility values between EQ-5D-5L and SF-6D of 0.165, which may appear somewhat surprising. This significant disagreement may be the consequence of instrument differences in terms of their health state classification systems (number and type of dimensions and levels included). The second possible cause may be a more than two-fold difference in the ranges of utility scales for both instruments (EQ-5D-5L: utilities from − 0.590 to 1.0, range 1.59; SF-6D: utilities from 0.296 to 1.0, range 0.704)—a consequence of two different valuation methods (time trade-off and standard gamble, respectively). The Bland–Altman analysis of instruments adjusted to the same scale (from 0 to 1) showed an increase of 0.395 in the mean difference in utility values between EQ-5D-5L and SF-6D. These results do not support the hypothesis that the main drivers of the differences between utility values estimated with different instruments were the differences in instrument ranges. After careful examination of the Bland–Altman plot for SF-6D (both unadjusted and adjusted), some form of inverted u-shape can be observed. This shape suggests that for mild and moderate health problems, SF-6D is more responsive than EQ-5D-5L. Contrary, for severe health problems, EQ-5D-5L is more responsive than SF-6D. The majority of the studied population seems to have mild or moderate health problems, the situation in which SF-6D appears to be the more responsive.

The major strength of our study is that the psychometric properties of EQ-5D-5L, EQ-5D-3L and SF-12 could be examined and compared against each other, since they were collected at the same time and within the same cohort. Another important strength is that we were able to use country-specific Polish utility algorithms to estimate and compare three EQ-based indices (two direct and one mapped) [21, 22, 24]. Past studies used crosswalk algorithms [13, 14] or only calculated an EQ-5D-5L index based on a direct value set [15]. Finally, another advantage of our research lies in its resistance to selection bias. Our results are based on data from respondents self-reporting diabetes in a sample representative of the entire population of Poland, in terms of gender, age and geographical region. Previous studies of EQ-5D-5L psychometric properties focused primarily on type 2 diabetes [12, 14, 15] and had a one-centre [13], three-centres [14] or regional [15] character.

Our study has some limitations. We included respondents based on self-reporting of diabetes without verified diagnoses using blood HbA1c, fasting plasma glucose level or through using data from medical records or registries. However, a similar approach may be found in epidemiological research into diabetes [38, 39] and the prevalence of diabetes in our study is in line with Polish research that is based on laboratory tests [40, 41]. Since our research had a cross-sectional nature, and we did not collect longitudinal data, we could not assess the responsiveness or test–retest reliability. Fortunately, this has been carried out by other authors [13–15]. We did not include a diabetes-specific instrument in the interview, which could have served as a standard anchor that best measures HRQoL in patients with diabetes. There are many disease-specific instruments used in diabetes studies, and none of them are indisputably considered to represent the gold standard of assessment. None of the five identified studies on EQ-5D-5L psychometric properties in diabetes used disease-specific instruments [10, 12–15]. Nevertheless, adding a diabetes-specific questionnaire to our comparisons would undoubtedly have deepened the current analysis. As described above, we presented the questionnaires in a fixed order, which might have generated a bias toward the lower response rate in EQ-5D-3L (given at the end) [42]. This was the reason for not assessing the feasibility. To some extent, we addressed potential memory effects, as the SF-12 questionnaire was presented between EQ-5D-5L and EQ-5D-3L. We only used records of respondents with complete HRQoL data and had to reject 8 (3.1%) patients with missing answers. This rejection percentage appears to be relatively low. Moreover, we avoided the necessity of missing scores imputation, which may always lead to bias. The lack of a country-specific value set forced us to estimate SF-6D values based on a UK algorithm developed by Brazier et al. [25], and the lack of a Polish algorithm for PCS-12 and MCS-12 constrained us into employing US norms from 2009 [43]. Most researchers have encountered similar problems, and the solutions we adopted are a standard approach. In simple terms, SF-12 and SF-6D have a lower number of country-specific algorithms than instruments from the EQ-5D family [44].

The golden rule of health-related quality of life research states that, when possible, the researcher should use both generic and disease-specific questionnaires. Our study shows that, in practice, both EQ-5D-5L and EQ-5D-3L are good candidates for the choice of generic instrument to be used in populations of patients with diabetes. The first format is characterised by a slightly lower ceiling effect and improved informativity in some dimensions. Nevertheless, the second format possesses some advantages within the self-care and usual activities dimensions. Another practical implication of our study is that countries lacking a directly-measured value set for EQ-5D-5L may still use mapped (cross-over) value sets in studies in diabetes, as we showed that both approaches produce valid measurements.

## Conclusions

In conclusion, evidence supports the EQ-5D-5L descriptive system and EQ-5D-5L index, based on a directly measured value set, as constituting valid generic HRQoL measures in respondents from the general population with self-reported diabetes. The EQ-5D-5L and EQ-5D-3L questionnaires showed clear psychometric advantages across different dimensions.

## Supplementary Information

**Additional file 1: Fig. 1**. Bland-Altman plots of EQ-5D-5L and (A) EQ-5D-3L, (B) SF-6D and (C) EQ VAS scores, after adjusting all utility instruments to the same scale (from 0 to 1).

## Data Availability

The datasets generated during the current study are available from the corresponding author on request.

## Abbreviations

ADDQoL: Audit of diabetes dependent quality of life (ADDQoL)
D-39: Diabetes-39 (D-39)
DCE: Discrete choice experiment
DHP: Diabetes health profile
DQOL: Diabetes quality of life measure
*H'*: Shannon index
HRQoL: Health-related quality of life
*J'*: Shannon evenness index
SC: Self-care
SD: Standard deviation
TTO: Time trade-off
UA: Usual activities
VAS: Visual analogue scale

## References

[1] Jing X, Chen J, Dong Y, Han D, Zhao H, Wang X (2018). Related factors of quality of life of type 2 diabetes patients: a systematic review and meta-analysis. Health Qual Life Outcomes.

[2] Zurita-Cruz JN, Manuel-Apolinar L, Arellano-Flores ML, Gutierrez-Gonzalez A, Najera-Ahumada AG, Cisneros-Gonzalez N (2018). Health and quality of life outcomes impairment of quality of life in type 2 diabetes mellitus: a cross-sectional study. Health Qual Life Outcomes.

[3] Meadows K, Steen N, McColl E, Eccles M, Shiels C, Hewison J (1996). The Diabetes Health Profile (DHP): a new instrument for assessing the psychosocial profile of insulin requiring patients–development and psychometric evaluation. Qual Life Res.

[4] The DCCT Research Group (1988). Reliability and validity of a diabetes quality-of-life measure for the diabetes control and complications trial (DCCT). Diabetes Care.

[5] Boyer JG, Earp JA. The development of an instrument for assessing the quality of life of people with diabetes. Diabetes-39. Med Care. 1997;35:440–53. 10.1097/00005650-199705000-00003 9140334

[6] Bradley C, Todd C, Gorton T, Symonds E, Martin A, Plowright R (1999). The development of an individualized questionnaire measure of perceived impact of diabetes on quality of life: the ADDQoL. Qual Life Res.

[7] Speight J, Reaney MD, Barnard KD (2009). Not all roads lead to Rome-a review of quality of life measurement in adults with diabetes. Diabetic Med.

[8] Janssen MF, Lubetkin EI, Sekhobo JP, Pickard AS (2011). The use of the EQ-5D preference-based health status measure in adults with Type 2 diabetes mellitus. Diabetic Med.

[9] Herdman M, Gudex C, Lloyd A, Janssen M, Kind P, Parkin D (2011). Development and preliminary testing of the new five-level version of EQ-5D (EQ-5D-5L). Qual Life Res.

[10] Janssen MF, Pickard AS, Golicki D, Gudex C, Niewada M, Scalone L (2013). Measurement properties of the EQ-5D-5L compared to the EQ-5D-3L across eight patient groups: a multi-country study. Qual Life Res.

[11] Feng YS, Kohlmann T, Janssen MF, Buchholz I (2021). Psychometric properties of the EQ-5D-5L: a systematic review of the literature. Qual Life Res.

[12] Matza LS, Boye KS, Stewart KD, Curtis BH, Reaney M, Landrian AS (2015). A qualitative examination of the content validity of the EQ-5D-5L in patients with type 2 diabetes. Health Qual Life Outcomes.

[13] Pattanaphesaj J, Thavorncharoensap M (2015). Measurement properties of the EQ-5D-5L compared to EQ-5D-3L in the Thai diabetes patients. Health Qual Life Outcomes.

[14] Koh D, Abdullah AM, Wang P, Lin N, Luo N. Validation of Brunei's Malay EQ-5D Questionnaire in patients with type 2 diabetes. PLoS One. 2016;11:e0165555. 10.1371/journal.pone.0165555 PMC5105939 27835652

[15] Sayah FA, Qiu W, Xie F, Johnson JA (2017). Comparative performance of the EQ-5D-5L and SF-6D index scores in adults with type 2 diabetes. Qual Life Res.

[16] Golicki D, Niewada M (2017). EQ-5D-5L Polish population norms. Arch Med Sci.

[17] Młyńczak K, Golicki D (2021). Validity of the EQ-5D-5L questionnaire among the general population of Poland. Qual Life Res.

[18] Golicki D, Niewada M (2015). General population reference values for 3-level EQ-5D (EQ-5D-3L) questionnaire in Poland. Pol Arch Med Wewn.

[19] Gandek B, Ware JE, Aaronson NK, Apolone G, Bjorner JB, Brazier JE, et al. Cross-validation of item selection and scoring for the SF-12 Health Survey in nine countries: results from the IQOLA Project. International Quality of Life Assessment. J Clin Epidemiol. 1998;51:1171–8. 10.1016/s0895-4356(98)00109-7 9817135

[20] Rencz F, Gulácsi L, Drummond M, Golicki D, Prevolnik Rupel V, Simon J (2016). EQ-5D in Central and Eastern Europe: 2000–2015. Qual Life Res.

[21] Golicki D, Dudzińska M, Zwolak A, Tarach JS (2015). Quality of life in patients with type 2 diabetes in Poland—comparison with the general population using the EQ-5D questionnaire. Adv Clin Exp Med.

[22] Golicki D, Jakubczyk M, Graczyk K, Niewada M (2019). Valuation of EQ-5D-5L health states in Poland: the first EQ-VT-based study in central and Eastern Europe. Pharmacoeconomics.

[23] Golicki D, Niewada M, Hout BV, Janssen MF, Pickard AS (2014). Interim EQ-5D-5L value set for Poland: first crosswalk value set in central and eastern Europe. Value Health Reg Issues.

[24] van Hout B, Janssen MF, Feng YS, Kohlmann T, Busschbach J, Golicki D (2012). Interim scoring for the EQ-5D-5L: mapping the EQ-5D-5L to EQ-5D-3L value sets. Value Health.

[25] Golicki D, Jakubczyk M, Niewada M, Wrona W, Busschbach JJ (2010). Valuation of EQ-5D health states in Poland: first TTO-based social value set in central and eastern Europe. Value Health.

[26] Brazier JE, Roberts J (2004). The estimation of a preference-based measure of health from the SF-12. Med Care.

[27] Johnson JA, Coons SJ (1998). Comparison of the EQ-5D and SF-12 in an adult US sample. Qual Life Res.

[28] Shannon CE, Weaver W (1998). The mathematical theory of communication.

[29] Fayers PM, Machin D, Fayers PM, Machin D (2007). Scores and measurements: validity, reliability, sensitivity. Quality of life: the assessment, analysis and interpretation of patient-reported outcomes.

[30] Golicki D, Niewada M, Buczek J, Karlińska A, Kobayashi A, Janssen MF (2015). Validity of EQ-5D-5L in stroke. Qual Life Res.

[31] Juniper EF, Guyatt GH, Jaeschke R, Spilker B (1996). Chapter 6: How to develop and validate a new health-related quality of life instrument. Quality of life and pharmacoeconomics in clinical trials.

[32] Mukaka MM (2012). Statistics corner: A guide to appropriate use of correlation coefficient in medical research. Malawi Med J.

[33] Bland JM, Altman DG (1986). Statistical methods for assessing agreement between two methods of clinical measurement. Lancet.

[34] Pan CW, Sun HP, Wang X, Ma Q, Xu Y, Luo N (2015). The EQ-5D-5L index score is more discriminative than the EQ-5D-3L index score in diabetes patients. Qual Life Res.

[35] Buchholz I, Janssen MF, Kohlmann T, Feng YS (2018). A systematic review of studies comparing the measurement properties of the three-level and five-level versions of the EQ-5D. Pharmacoeconomics.

[36] Gamst-Klaussen T, Gudex C, Olsen JA (2018). Exploring the causal and effect nature of EQ-5D dimensions: an application of confirmatory tetrad analysis and confirmatory factor analysis. Health Qual Life Outcomes.

[37] Poder TG, Carrier N, Kouakou CRC (2020). Quebec health-related quality-of-life population norms using the EQ-5D-5L: decomposition by sociodemographic data and health problems. Value Health.

[38] Li HL, Fang J, Zhao LG, Liu DK, Wang J, Han LH (2020). Personal characteristics effects on validation of self-reported type 2 diabetes from a cross-sectional survey among Chinese adults. J Epidemiol.

[39] Yuan X, Liu T, Wu L, Zou ZY, Li C. Validity of self-reported diabetes among middle-aged and older Chinese adults: the China Health and Retirement Longitudinal Study. BMJ Open 2015;5:e006633. 10.1136/bmjopen-2014-006633 PMC4401856 25872937

[40] Rutkowski M, Bandosz P, Czupryniak L, Gaciong Z, Solnica B, Jasiel-Wojculewicz H (2014). Prevalence of diabetes and impaired fasting glucose in Poland–the NATPOL 2011 Study. Diabetic Med.

[41] Zatońska K, Ilow R, Regulska-Ilow B, Różańska D, Szuba A, Wołyniec M (2011). Prevalence of diabetes mellitus and IFG in the prospective cohort 'PONS' study—baseline assessment. Ann Agric Environ Med.

[42] Poder TG, Fauteux V, He J, Brazier JE (2019). Consistency between three different ways of administering the short form 6 dimension version 2. Value Health.

[43] Maruish ME, editors. User’s manual for the SF-12v2 Health Survey. 3rd ed. Lincoln, RI: QualityMetric Incorporated; 2012.

[44] Devlin NJ, Brooks R (2017). EQ-5D and the EuroQol group: past, present and future. Appl Health Econ Health Policy.

