# Supplementary material for: Validity of EQ-5D-5L health-related quality of life questionnaire in self-reported diabetes: evidence from a general population survey
Source: Health Qual Life Outcomes. 2021 May 5;19:138. doi: 10.1186/s12955-021-01780-2 (PMC8097836; doi:10.1186/s12955-021-01780-2)

Suppl. Fig. 1 Bland-Altman plots of EQ-5D-5L and (A) EQ-5D-3L, (B) SF-6D and (C) EQ VAS scores, after adjusting all utility instruments to the same scale (from 0 to 1)

A

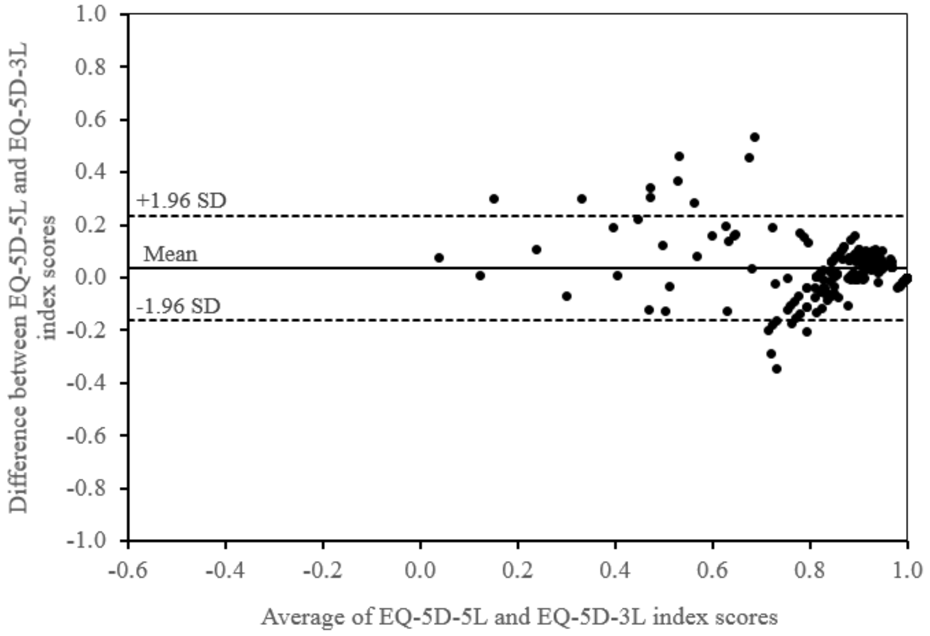

B

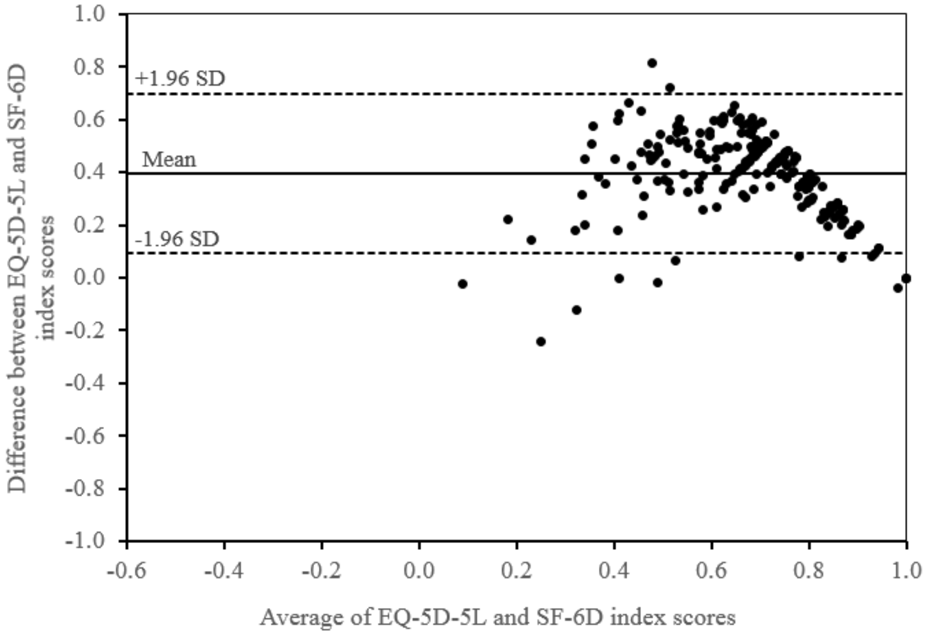

C

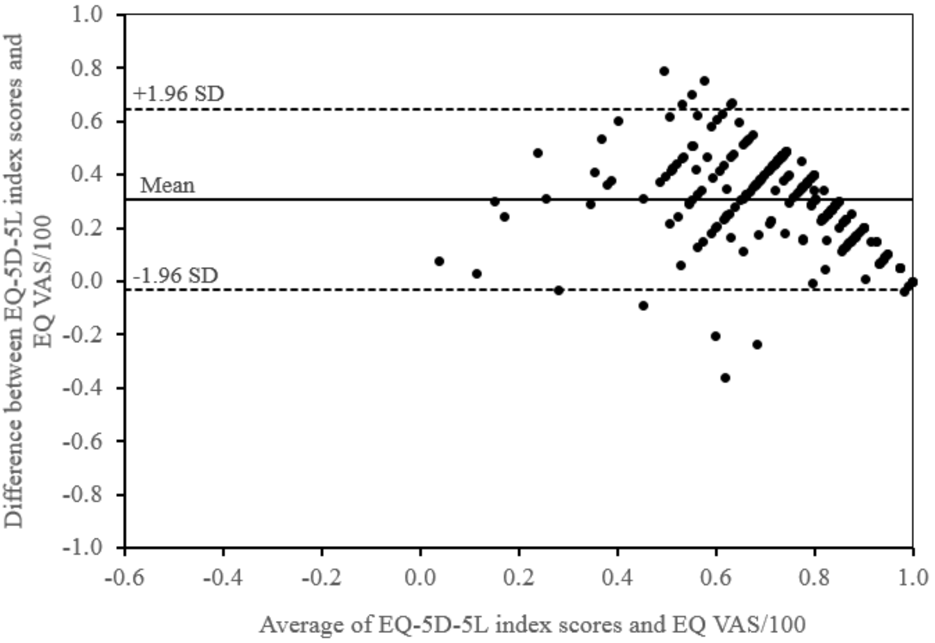

Supplement: Supplementary file 1 — Additional file 1: Fig. 1. Bland-Altman plots of EQ-5D-5L and (A) EQ-5D-3L, (B) SF-6D and (C) EQ VAS scores, after adjusting all utility instruments to the same scale (from 0 to 1). [file 12955_2021_1780_MOESM1_ESM.pdf]
